# Hydrophilic Polymer Embolism and Silent Brain Injury: An Underexplored Connection

**DOI:** 10.1007/s00270-025-04186-5

**Published:** 2025-09-10

**Authors:** Pablo Albiña-Palmarola, Hans Henkes

**Affiliations:** 1 Neuroradiologische Klinik, Kopf- Und Neurozentrum, Klinikum Stuttgart, Kriegsbergstraße 60, 70174 Stuttgart, Germany; 2 Medizinische Fakultät, Universität Duisburg-Essen, Hufelandstraße 55, 45122 Essen, Germany

The recent scoping review by Parillo and colleagues provides a structured overview of hydrophilic polymer embolism (HPE), drawing attention to a complication of endovascular procedures that is still underestimated in daily practice [1]. By collating evidence from pathology studies, case series, and case reports, the authors show that polymer coating delamination and embolization can affect multiple vascular territories, often with delayed or nonspecific consequences. Their review highlights a phenomenon recognized for over a decade but still insufficiently integrated into clinical reasoning. Autopsy studies summarized reported HPE in 13–23% of cases, while targeted thrombus analyses found polymer fragments in up to 86% of patients undergoing percutaneous or neurovascular interventions. These findings indicate that device-related embolization is not rare but frequently overlooked due to subtle imaging features and the lack of routine histological confirmation. The wide range of manifestations—from acute infarcts to delayed granulomatous reactions such as nonischemic cerebral enhancing (NICE) lesions—underscores both the diagnostic challenges and clinical heterogeneity of HPE.

A critical and still unresolved issue is the potential role of HPE in silent ischemic brain injury. According to some studies, as many as half of all neurointerventions are followed by silent DWI lesions, the mechanisms of which remain only partly understood [2]. Traditionally, air and thromboembolism have been invoked, but no single mechanism explains their high frequency. The clinicopathological evidence compiled by Parillo et al., when considered alongside more recent experimental and clinical data, raises the possibility that hydrophilic polymer debris could contribute to these lesions. Our own observations are compatible with this hypothesis. Using attenuated total reflection Fourier transform infrared spectroscopy, we identified polyvinylpyrrolidone delamination from a specific microcatheter during routine withdrawal, with polymer particles confirmed both in vitro and in clinical material [3]. In a separate cohort of 404 patients with unruptured intracranial aneurysms undergoing flow diversion, use of the same type of microcatheter was independently associated with a threefold increase in silent postprocedural DWI lesions compared with other devices, even after adjustment for procedural and patient variables [4]. These findings, although still unproven, could be explained by unexpected HPE, consistent with broader clinicopathological evidence that polymer emboli are commonly incorporated into thrombi obtained during mechanical thrombectomy or percutaneous coronary intervention.

The possible link between HPE and silent ischemic lesions carries substantial implications. Silent infarcts are not clinically trivial; they have been associated with progressive cognitive decline, particularly in aging populations already at risk [5]. Moreover, delayed inflammatory responses such as NICE lesions may require immunosuppressive therapy and indicate that the consequences of HPE can extend well beyond the immediate procedural period. If coating embolization proves to be a driver of cumulative cerebral microinjury, it may represent a preventable contributor to vascular cognitive impairment.

The scoping review also raises broader regulatory and technical questions. Current frameworks provide manufacturers considerable autonomy in defining coating specifications and interpreting particulate testing, leaving variability in safety standards. Moving forward, systematic evaluation of device-specific embolic potential, prospective imaging studies that correlate silent lesions with device use and long-term cognitive follow-up is necessary. In parallel, refinements in coating technologies and procedural techniques may mitigate risk.

Parillo et al. have assembled a valuable reference point for this discussion, but further work is needed to establish the prevalence, mechanisms, and clinical impact of HPE. The interventional community should now move from anecdotal recognition toward systematic investigation. Understanding and preventing device-related brain injury is not only a technical challenge but a responsibility, given the scale of modern neurointerventional practice and its long-term implications for patients.
